# The double homeobox a pseudogene 8 accelerates cell proliferation, migration, and invasion in colon cancer

**DOI:** 10.1080/21655979.2022.2053802

**Published:** 2022-03-24

**Authors:** Shengxun Mao, Zhaohong Mo, Runxin Wu, Bin Lai, Zhiyong Zhou, Yi Song, Xi Ouyang, Xingen Zhu

**Affiliations:** a Department of Gastrointestinal Surgery, The Second Affiliated Hospital of Nanchang University, Nanchang, China; b Department of Hepatobiliary Surgery, The Third Affiliated Hospital of Sun Yat-sen University, Guangzhou, China; c Zhongshan School of Medicine, Sun Yat-Sen University, Guangzhou, China; d Department of Neurosurgery, The Second Affiliated Hospital of Nanchang University, Nanchang, China

**Keywords:** DUXAP8, invasion, migration, proliferation, colon cancer

## Abstract

Double homeobox A pseudogene 8 (DUXAP8) is a known tumor promoter in several malignancies. Nonetheless, its function in colon cancer (CC) is indefinite. Herein, we explored the significance of DUXAP8 and its underlying mechanism in CC. Our data indicated that DUXAP8 was upregulated in CC, and it was related to advanced stages and lymph node metastases. Based on our Kaplan-Meier survival analysis, elevated DUXAP8 expression resulted in shorter patient overall survival (OS). Conversely, DUXAP8 silencing strongly suppressed cellular proliferation, migration and invasion *in vitro*. Based on our western blot analysis, DUXAP8 deficiency strongly inhibited the epithelial–mesenchymal transition (EMT) *in vitro*. Alternately, DUXAP8 overexpression accelerated cellular proliferation migration and invasion in CC. Finally, silencing DUXAP8 prevented tumorigenesis in a mouse xenograft model *in vivo*. Collectively, our results demonstrated that DUXAP8 regulates the occurrence and advancement of CC, and may serve as a regulatory hub for this disease.

## Graphical abstract

**Figure:**
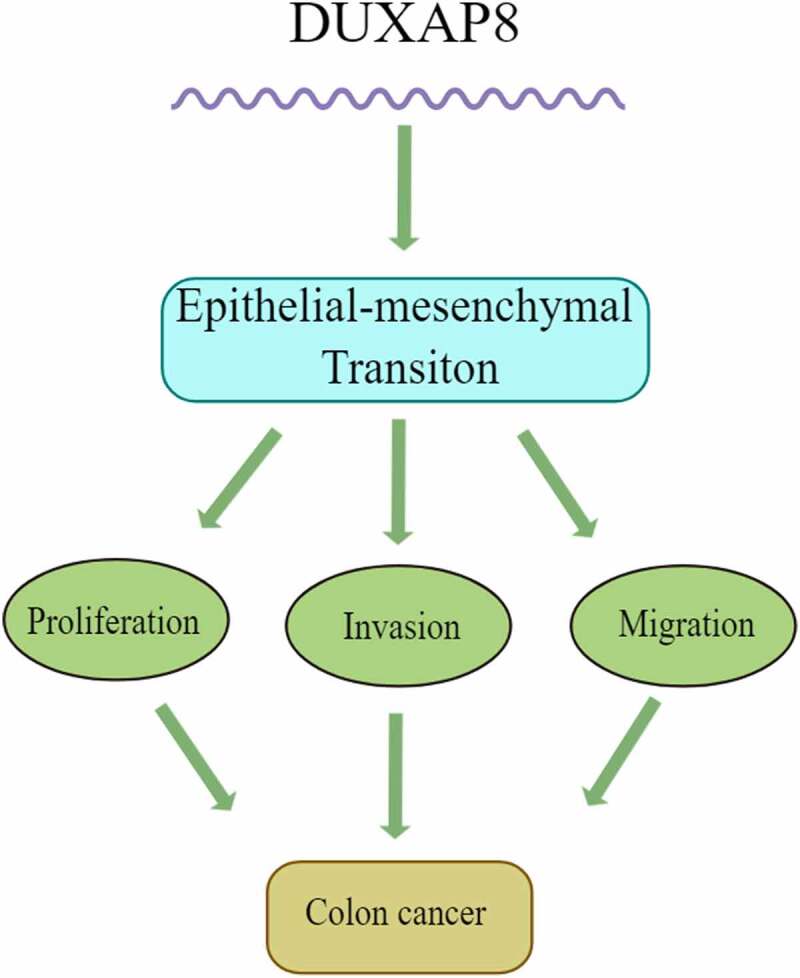

## Introduction

Colon cancer (CC) is the third ranking carcinoma in the world, with 1,880,000 new cases, and 915,000 deaths in 2020 alone^1^. Lately, the overall survival (OS) rate of CC has improved tremendously, due to new treatment regimens, including, preoperative chemotherapy and targeted treatment. However, 50% of CC patients still experience recurrence and metastasis following treatment^2^. Therefore, identifying the pivotal molecular mechanism regulating tumor occurrence and progression in CC is crucial for the screening of novel targeted therapeutics that enhance prognosis of these patients.

Long non-coding RNAs (lncRNAs) have extensive functions, and participate in multiple cellular processes, such as, tumorigenesis and progression^3,4^. LncRNA FAM225A is upregulated in nasopharyngeal carcinoma (NPC), and its overexpression promotes NPC tumorigenesis and metastasis^5^. Another important lncRNA is LncRNA GLCC1, which is reported to promote colorectal carcinogenesis and glucose metabolism via stabilization of c-Myc^6^. Being a special class of lncRNAs, pseudogenes were initially regarded as nonfunctional genomic relics of functional genes. Current research demonstrated that pseudogene-derived RNAs in fact modulate human cancer progression via other mechanisms, such as, serving as competing endogenous RNAs (ceRNAs), and as competitors for RBP or translational machinery^7,8^. Recent investigations suggested that DUXAP10 functions as an oncogene to promote cell proliferation via the PI3K/AKT pathway in hepatocellular carcinoma (HCC)^9^. HMGA1P6 is activated by MYC, and promotes cell malignancy by serving as a ceRNA in ovarian cancer^10^. Double homeobox A pseudogene 8 (DUXAP8) is a retrotransposed pseudogene, encoded by the human double homeobox A (DUXA), residing in 22q11, and carrying a length of 2107 bp^11^. DUXAP8 serves a critical function in numerous human malignant tumors, such as, HCC^12^, glioma^13^, and gastric cancer^14^. A recent study suggested that DUXAP8 accelerates cellular proliferation of papillary thyroid carcinoma via the ceRNAs mechanism^15^. In triple negative breast cancer, DUXAP8 is activated by the YY1 transcription factor, and it promotes cellular proliferation of cancer cells via upregulation of SAPCD2^16^. However, the function of DUXAP8 in CC is still undetermined, and requires additional investigation.

Our goal here was to assess the DUXAP8 expression in CC, elucidate its potential regulation of cellular proliferation, migration and invasion, and clarify the underlying mechanism. To this end, we examined DUXAP8 expression in CC tissues and cells. In addition, we investigated the relation between DUXAP8 and pathologic characteristics. Next, we examined the DUXAP8-mediated modulation of cellular proliferation, migration and invasion. Mechanistically, we evaluated vimentin and e-cadherin levels under DUXAP8 overexpression and deficiency, to better understand its influence on the epithelial–mesenchymal transition (EMT) *in vitro*. Our analysis identified a new function for DUXAP8, which may provide a novel insight into the treatment of CC patients.

## Materials and methods

### Tissue samples

Between March 2014 and September 2015, we obtained the surgical samples of 60 CC patients from the Second Affiliated Hospital of Nanchang University (Nanchang, China). This included CC tissues and corresponding adjoining non-tumor colon mucosa tissues (NTT). CC disease was diagnosed via pathology, and none of the subjects received chemo- or radiotherapy prior to surgery. Our work received ethical approval from our hospital (2019 No. 100). The resected samples were immediately stored at –80°C. The patient clinicopathologic profiles are summarized in Supplemental Table 1.

## Cell culture

The cell lines HCT116, SW480, LoVo, SW620, HT29, and NCM460 were obtained from the Chinese Academy of Science. All cells were maintained in DMEM (Gibco, USA) with 10% of fetal bovine serum (FBS; Invitrogen, Carlsbad, USA), 100 U/ml penicillin, and 100 mg/ml streptomycin (Beyotime Biotechnology, Shanghai, China) in a humid chamber with 5% CO_2_, and at 37°C.

## Quantitative real-time PCR (qRT-PCR)

Total RNA was retrieved with Trizol® (Invitrogen, Carlsbad, USA), and cDNA synthesis was done with the PrimeScript™ RT Master Mix (Takara Biomedical Technology Co., Ltd. Beijing). Then, qRT-PCR analysis was carried out with TB Green™ Premix Ex Taq™ II in an ABI PRISM® 7500 FAST System (Applied Biosystems, Foster City, CA, USA), as per kit directions. Briefly, the reaction conditions were as follows: denaturation at 95°C for 5 min; then 35 cycles of 95°C for 10s, 60°C for 30s, and 72°C for 30s. GAPDH was employed as the housekeeping gene. The relative DUXAP8 expressions were obtained with the 2-ΔΔCt method^16^. The DUXAP8 primers were as follow: 5’-AGGATGGAGTCTCGCTGTATTGC-3’ (forward) and 5’-GGAGGTTTGTTTTCTTCTTTTTT-3’ (reverse). The GAPDH primers were as follow: 5’-GGGAGCCAAAAGGGTCAT-3’ (forward) and 5’-GAGTCCTTCCACGATACCAA-3’ (reverse).

## Cell transfection

The overexpression vectors, shRNA vectors, and relevant negative control vector were manufactured by TSINHKE Biological Technology (Guangzhou, China). Transfection was performed using Lipofectamine 3000 (Invitrogen, Carlsbad, USA). Briefly, 1 × 10^5^ cells were placed on a 6-well plate, and incubated until 70% confluency. The transfection reagent and vector were mixed after dilution with serum‐free medium (SFM). Next, the cells were cultured with the mixture at 37°C for 12 hours, after which the medium was refreshed. After 24 hours, the cells were harvested for RNA extraction. The sequences of the sh-DUXAP8 were as follows: sh-DUXAP8-1:5’-CACCAAGATAAAGGTG GTTTCCACAAGAACGAATTCTTGTGGAAACCACCTTTATC-3’; shDUXAP8-2: 5’-CACCGCA GCATACTTCAAATTCACAGCAAACGAATTTGCTGTGAATTTGAAGTATGCTG-3’; sh-NC: 5’- CCGGGTTCTCCGAACGTGTCACGTCTCGAGACGTGACACGTTCGGAGAACCTTTTTG-3’.

## Cell proliferation

The Cell Counting kit-8 (CCK-8) was employed for the CC cellular proliferation capacity assessment. Briefly, 1 × 10^3^ cells were placed per well of a 96-well plate. Subsequently, we introduced the CCK‐8 solution, and evaluated absorbance at 450 nm following a 2 h incubation at 37°C.

## Migration and invasion assays

The migration and invasion chamber systems were purchased from Corning (NY 14831 USA), and the experiments were performed as described previously^17^. In short, 2 × 10^5^ cells were placed into the top chamber of inserts, with or without pre-coated Matrigel, and incubated with SFM. In the bottom chamber, we introduced 10% FBS containing culture medium to induce chemotaxis. Following a 24 h incubation, the cells that traveled to the bottom chamber underwent fixation in 4% paraformaldehyde, and staining with 0.1% crystal violet. Lastly, the invading cells were counted under a fluorescent microscope (Carl Zeiss AG).

## Wound healing assay

Overall, 1 × 10^5^ cells were placed per well in a 6-well plate, and incubated until 80–90% confluency. Then, a 10 μl pipette tip was employed to generate a scratch, and the migration distance was measured under a phase-contrast microscope (Carl Zeiss AG), after 48 h of culture.

## Western blot analysis

Total protein was retrieved with RIPA buffer and protease inhibitors, and protein quantification was done with the BCA protein Assay Kit (Millipore, Darmstadt, Germany). Next, 10ug proteins underwent separation via SDS-PAGE, then transfer to a polyvinylidene fluoride membrane (Millipore, Darmstadt, Germany), and subsequent treatments as follows: sealing with 5% nonfat milk, incubation with specific primary and secondary antibodies, and lastly, quantification via chemiluminescence. All primary antibodies were diluted 1:1000 and were acquired from Cell Signaling Technology (Danvers, MA, USA). The employed antibodies were as follows: anti-GAPDH (#5174), anti-E-cadherin(#3195), and anti-vimentin (#5741). The protein band intensity was visualized and quantifed via the ImageJ software. GAPDH expression was employed as internal reference.

## *In vivo* tumor growth experiments

Five to six week old BALB/c nude mice were selected (n = 4 per group). Our animal protocols received ethical approval from our institution. HCT116 and HT29 cells (1x10^7^ cells/ml) were administered into the posterior sides of mice in various groups (shRNA-DUXAP8 and control). Following two weeks of icubation, we euthanized the mice, and evaluated their tumor volumes.

## Statistical analysis

All data analyses were carried out with the SPSS 22.0 software (SPSS, Inc, IL, USA). All experiments were conducted in triplicates. Inter-group comparisons were done with the Student’s t test. We plotted OS curves via the Kaplan–Meier curve analysis. P < 0.05 was set as the significance threshold.

## Results

In the last decade, growing evidences suggested that the pseudogene-derived RNAs serve a crucial role in CC progression. In particular, DUXAP8 is upregulated and promotes cellular proliferation in papillary thyroid carcinoma and triple negative breast cancer^15,18^. Nevertheless, the DUXAP8 significance in CC remains unclear, and requires additional investigation. Herein, we elucidated the DUXAP8-mediated regulation of cellular proliferation, migration, and invasion. We also clarified potential mechanisms, in order to provide a novel insight into the treatment of CC patients.

## DUXAP8 levels are elevated in CC, and it is correlated with poor outcome

**Figure 1.**
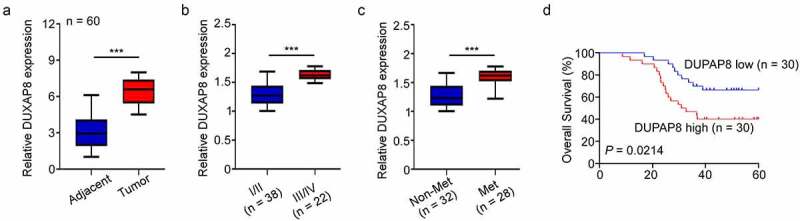
Elevation in DUXAP8 levels are connected to poor cancer prognosis in colon cancer (CC) patients, as evidenced by qRT-PCR. (a) enhanced DUXAP8 levels in CC tissues versus corresponding non-tumor samples. (b) elevated DUXAP8 expression in patients at III/IV stages versus I/II stages. (c) elevated DUXAP8 levels in patients with lymphatic metastasis than without lymphatic metastasis. (d) CC patients expressing higher DUXAP8 levels experience shorter overall survival (OS) than patients with low DUXAP8 levels. ***P < 0.001.

To delineate the profile and significance of DUXAP8 in CC, 60 pairs of CC tissues and corresponding adjoining NTT were included. Based on our qRT-PCR results, the DUXAP8 levels were markedly elevated in CC tissues, relative to NTT (Figure 1a). We, next, classified CC patients into two groups, based on their stage and lymph node metastatic statuses. As shown in Figure 1b-c, DUXAP8 upregulation was strongly association with advanced stages and lymph node metastases. Moreover, our Kaplan-Meier OS analysis revealed shorter OS rates in the CC patients with elevated DUXAP8 expression, relative to controls (Figure 1d).

## DUXAP8 knockdown suppressed CC cell proliferation

**Figure 2.**
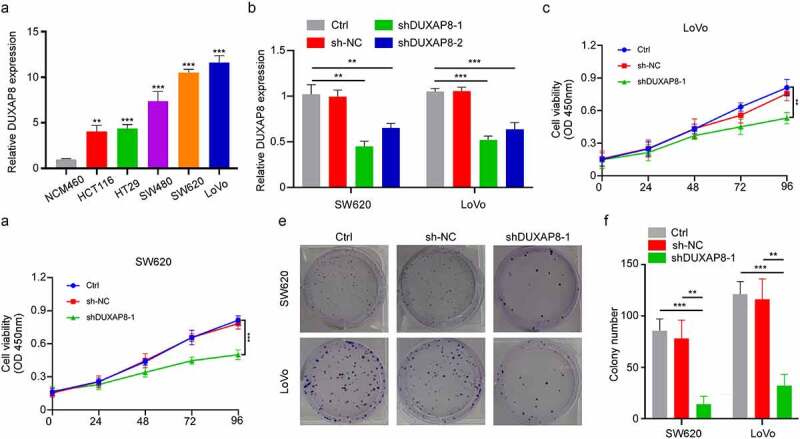
DUXAP8 knockdown inhibits cell proliferation of colon cancer (CC) cells, as evidenced by qRT-PCR. (a) markedly elevated DUXAP8 levels in five CC cell lines versus healthy colonic epithelial cells. (b) descended DUXAP8 levels following transfection with the sh-DUXAP8. (c-d) DUXAP8 deficiency in SW620 and LoVo cells significantly inhibits cellular proliferation. (e) DUXAP8 deficiency in SW620 and LoVo cells suppresses colony formation, relative to controls. (f) colonies were counted in (E). **P < 0.01, ***P < 0.001.

Given that DUXAP8 was highly expressed in CC patients, we further examined its influence in CC cellular proliferation. Using qRT-PCR, we demonstrated that the DUXAP8 levels were significantly increased in CC cell lines versus colon epithelial cells (Figure 2a). Next, we incorporated shRNA to knockdown DUXAP8 expression in the SW620 and LoVo cells, and assessed its subsequent biological effects (Figure 2b). Our CCK8-assay indicated that the DUXAP8 knockdown markedly suppressed cellular proliferation (Figure 2c–d). Moreover, as depicted in Figure 2e-f, DUXAP8 knockdown also prevented colony formation, relative to controls.

## DUXAP8 knockdown inhibited CC migration and invasion

**Figure 3.**
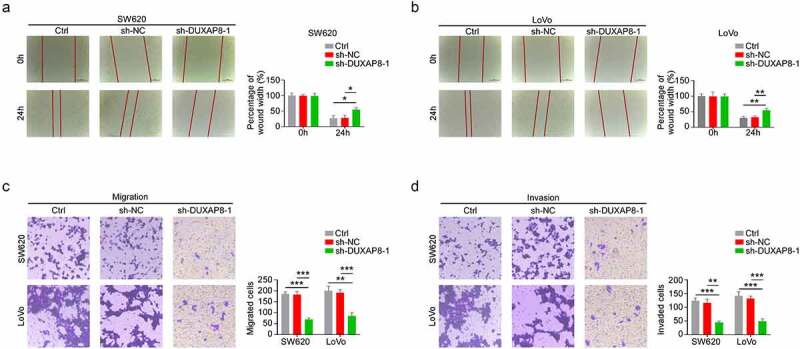
DUXAP8 deficiency prevents colon cancer (CC) migration and invasion. (a-b) DUXAP8 knockdown significantly enhances the relative wound width, relative to controls. (c-d) DUXAP8 knockdown strongly diminished migration and invasion, relative to controls. *P < 0.05, **P < 0.01, ***P < 0.001.

We, next, elucidated whether DUXAP8 affects migration and invasion of CC cells. Based on our wound healing assay results, the relative wound width was significantly enhanced in the sh-DUXAP8 group, thereby suggesting that the migration of CC was inhibited after DUXAP8 knockdown (Figure 3a-b). Similarly, using transwell assays, we demonstrated that, compared to the control group, the migration and invasion were drastically suppressed in the sh-DUXAP8 group, relative to controls (Figure 3c-d). Collectively, these results suggested that DUXAP8 knockdown suppresses both CC migration and invasion.

## DUXAP8 overexpression promoted CC cellular proliferation

**Figure 4.**
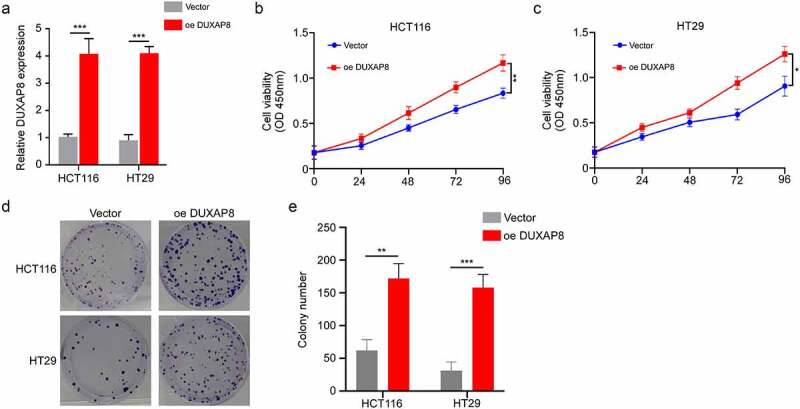
DUXAP8 incorporation accelerates colon cancer (CC) cell proliferation. (a) structured pcDNA 3.1-DUXAP8 incorporation significantly enhanced DUXAP8 levels. (b-c) DUXAP8 overexpression markedly increased cellular proliferation. (d) following pcDNA 3.1-DUXAP8 transfection, colonies were more and larger than the control groups. (e) colonies were counted in (D). **P < 0.01, ***P < 0.001.

In previous experiments, we observed that DUXAP8 expression was reduced in HCT116 and HT29 CC cells. Hence, we overexpressed DUXAP8 in those cells, and evaluated its influence on cellular proliferation. We incorporated cells with the structured pcDNA 3.1-DUXAP8 to enhance DUXAP8 levels. Using qRT-PCR, we revealed that the pcDNA 3.1-DUXAP8 incorporation significantly elevated DUXAP8 levels within cells (Figure 4a). Based on our CCK8-assay, the cellular proliferation was markedly enhanced following DUXAP8 overexpression, relative to controls (Figure 4b-c). Furthermore, increased DUXAP8 levels accelerated colony formation, relative to controls (Figure 4d-e).

## DUXAP8 overexpression accelerated CC migration and invasion

**Figure 5.**
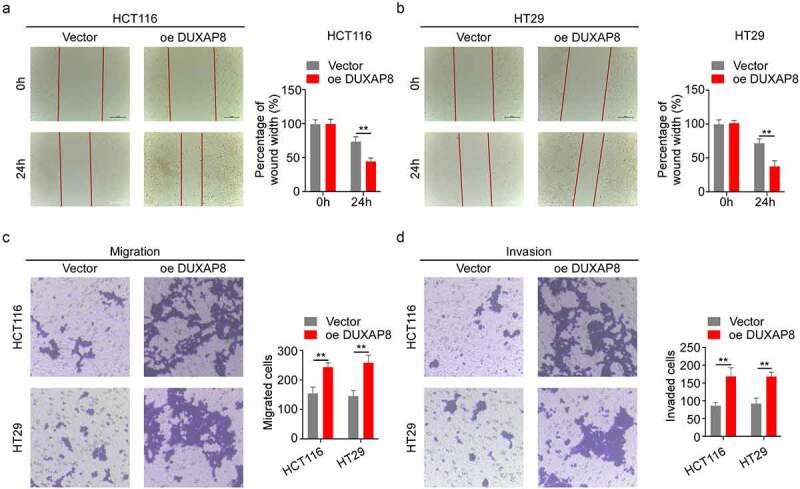
DUXAP8 incorporation accelerates colon cancer (CC) migration and invasion. (a-b) DUXAP8 overexpression drastically decreased the relative wound width. (c-d) DUXAP8 overexpression markedly enhanced migration and invasion versus controls. **P < 0.01.

We, next, assessed the impact of DUXAP8 overexpression on CC migration and invasion. The relative wound width was drastically diminished in the oe-DUXAP8 group, which suggested that the CC migration was markedly increased after DUXAP8 overexpression (Figure 5a-b). Similarly, the transwell assays revealed that, compared to the control group, the oe-DUXAP8 group exhibited accelerated migration and invasion (Figure 5c-d).

## DUXAP8 modulated CC tumorigenesis *in vivo*

**Figure 6.**
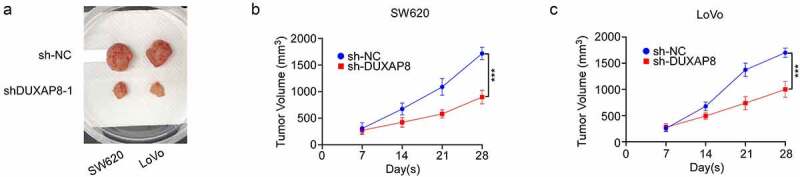
DUXAP8 levels modulated tumorigenesis of colon cancer (CC) *in vivo*. (a) DUXAP8 deficiency markedly decreases tumor volume. (b-c) tumor growth curves. tumor size was calculated every other day for 7 days post injection.

Given that we already established that DUXAP8 promotes CC cellular proliferation *in vitro*. We, next, examined whether DUXAP8 also accelerates CC growth in a mouse xenograft model *in vivo*. Two weeks after CC cell administration, the tumors volume was measured. Based on our analysis, DUXAP8 knockdown remarkably decreased tumor volume, relative to controls (Figure 6a-c). These data indicated that DUXAP8, indeed, modulated CC tumorigenesis *in vivo*.

## DUXAP8 was essential for EMT

**Figure 7.**
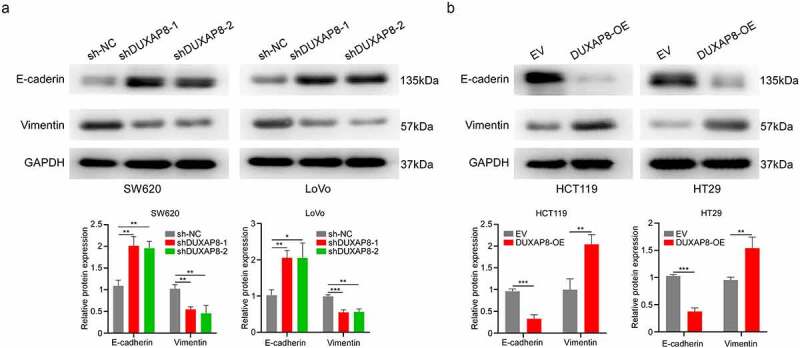
DUXAP8 is critical for epithelial–mesenchymal transition (EMT). (a) DUXAP8 knockdown results in an upregulation of e‐cadherin expression, and suppression of vimentin levels. (b) DUXAP8 overexpression leads to the downregulation of e‐cadherin expression, and elevation of vimentin levels. **P < 0.01, ***P < 0.001.

In order to elucidate the underlying mechanism behind the DUXAP8-mediated regulation of CC migration and invasion, we investigated the EMT-related protein expressions. The e‐cadherin levels were markedly increased, whereas, vimentin levels were strongly suppressed after DUXAP8 knockdown, as evidenced by western blot (Figure 7a). In contrast, the e‐cadherin levels were strongly suppressed, whereas, the vimentin levels were enhanced in the oe-DUXAP8 group versus controls (Figure 7b). Collectively, these results indicated that DUXAP8 induces EMT in CC.

## Discussion

Prior studies suggested that pseudogene serves an essential function in promoting tumor occurrence in breast cancer^19^, non-small cell lung cancer^11^, CC^20^ and pancreatic carcinoma^21^. In this study, we demonstrated that DUXAP8 is a principal modulator of tumor occurrence and advancement in CC. Based on our analysis, DUXAP8 levels were markedly elevated in CC tissues, and they were strongly associated with the pathological stage and lymph node metastatic status of CC. Furthermore, the OS curves indicated that the higher expression of DUXAP8 represented worse OS in CC patients. Collectively, these results suggested that DUXAP8 serves as an oncogene, and is linked to poor prognosis in CC patients.

Pseudogene-derived RNAs are critical for various aspects of tumor biology in multiple tumors, including, tumorigenesis, apoptosis, and invasion^22,23^. Decreased pseudogene-derived RNASFTA1P suppresses cellular proliferation in gastric cancer^24^. Lian et al. reported that the pseudogene-derived RNADUXAP10 is highly expressed in colorectal cancer tissues. Furthermore, several functional and mechanistic investigations revealed that DUXAP10 promotes cellular proliferation and tumorigenesis via silencing of the p21 and PTEN gene expressions^25^. Recently, increasing studies are investigating the DUXAP8 role in tumor. Wei et al. demonstrated marked upregulation in DUXAP8 levels in HCC, which, in turn, triggers cellular proliferation, migration, and invasion of HCC cells via regulation of the miR-422a/PDK2 axis^26^. Another study reported that DUXAP8 downregulates miR-126 expression, which, in turn, promotes the renal cell carcinoma progression^27^. In our investigation, DUXAP8 knockdown strongly suppressed CC cellular proliferation, migration, and invasion. In contrast, DUXAP8 overexpression markedly enhanced cellular proliferation, migration, and invasion, similar to prior studies involving HCC^26^. Further analysis indicated that the transplanted CC growth was significantly abrogated after DUXAP8 knockdown in the xenograft experiment *in vivo*.

EMT is strongly associated with tumor invasion and metastasis in numerous cancers, including, colon^28^, gastric^29^, and prostate cancers^30^. It was previously demonstrated that several lncRNAs modulate EMT to regulate tumor metastasis^31,32^. Following EMT development, a polarized epithelial cell assumes the phenotype of a mesenchymal cell via multiple biochemical alterations^33^. In this study, DUXAP8 knockdown inhibited vimentin, and raised e-cadherin expression in CC cells. Alternately, DUXAP8 overexpression enhanced vimentin levels, while downregulating e-cadherin expression in CC cells. Our study demonstrated that DUXAP8 accelerated CC metastasis by facilitating EMT. We also demonstrated that DUXAP8 levels were markedly elevated in CC, and induced CC cellular proliferation, migration, and invasion. Nevertheless, it is unclear whether other downstream genes or regulatory mechanisms are involved in this process. Therefore, further investigations are warranted to clarify the functions of DUXAP8, and identify underlying mechanisms that promote the development of CC.

## Conclusion

In summary, we demonstrated that DUXAP8 was significantly upregulated in CC. Additional analyses indicated that DUXAP8 accelerated CC cellular proliferation, migration, and EMT. Our work also indicated that CC patients with elevated DUXAP8 expression experience worse prognosis. Based on these evidences, DUXAP8 influences the occurrence and progression of CC, and may, therefore, serve as a regulatory hub for this disease.

## Supplementary Material

Supplemental Material

## Highlights

1. DUXAP8 is highly expressed in CC.
2. DUXAP8 may promote the proliferation, migration and invasion of CC cells.
3. DUXAP8 was required for EMT.
