# Supplementary material for: The double homeobox a pseudogene 8 accelerates cell proliferation, migration, and invasion in colon cancer
Source: Bioengineered. 2022 Mar 24;13(4):8164–73. doi: 10.1080/21655979.2022.2053802 (PMC9161926; doi:10.1080/21655979.2022.2053802)
Supplement: Supplemental Material [file KBIE_A_2053802_SM7640.doc]

Supplemental Table 1.The correlation of DUXAP8 expression with clinicopathological parameters of colon cancer patients

| Variables | Clinicopathological  parameters | Patient  frequency (n=60) | DUXAP8 | P value |
| --- | --- | --- | --- | --- |
|  |  |  | Low High |  |
| Gender | Male | 34 | 16 18 | 0.794 |
|  | Female | 26 | 14 12 |  |
| Age | <60 | 39 | 18 21 | 0.588 |
|  | ≥60 | 21 | 12 9 |  |
| Tumor stage | T1-T2 | 38 | 26 12 | 0.000 |
|  | T3-T4 | 22 | 4 18 |  |
| Metastasis status | M0 | 41 | 26 15 | 0.006 |
|  | M1 | 19 | 4 15 |  |
| Lymph node status | N (-) | 32 | 23 9 | 0.000 |
|  | N (+) | 28 | 7 21 |  |
